# *Dec2* attenuates autophagy in inflamed periodontal tissues

**DOI:** 10.1002/iid3.389

**Published:** 2020-12-03

**Authors:** Shunichi Oka, Xiaoyan Li, Fuyuki Sato, Fengzhu Zhang, Nitesh Tewari, Chongchong Chen, Liangjun Zhong, Makoto Makishima, Yi Liu, Ujjal K. Bhawal

**Affiliations:** ^1^ Department of Anesthesiology Nihon University School of Dentistry Tokyo Japan; ^2^ Division of Immunology and Pathology, Dental Research Center Nihon University School of Dentistry Tokyo Japan; ^3^ Laboratory of Tissue Regeneration and Immunology and Department of Periodontics, Beijing Key Laboratory of Tooth Regeneration and Function Reconstruction Capital Medical University School of Stomatology Beijing People's Republic of China; ^4^ Pathology Division Shizuoka Cancer Center Shizuoka Japan; ^5^ Department of Anesthesiology Nihon University School of Dentistry at Matsudo Chiba Japan; ^6^ Division of Pedodontics and Preventive Dentistry, Centre for Dental Education and Research All India Institute of Medical Sciences New Delhi India; ^7^ Department of Stomatology Hangzhou Normal University Hangzhou People's Republic of China; ^8^ Division of Biochemistry, Department of Biomedical Sciences Nihon University School of Medicine Tokyo Japan; ^9^ Department of Disaster Medicine and Dental Sociology Kanagawa Dental University Yokosuka Japan; ^10^ Department of Biochemistry and Molecular Biology Nihon University School of Dentistry at Matsudo Chiba Japan

**Keywords:** autophagy, Dec2, ERK/mTOR pathway, knock‐out animal model, *P. gingivalis*, periodontal inflammation

## Abstract

**Introduction:**

Transcriptional regulation of autophagy depends on the transcription factors coordinated inflammatory feedback mechanism. Here, we provide a comprehensive functional characterization of periodontal ligament fibroblasts (PDLFs) treated with *Porphyromonas gingivalis* lipopolysaccharide (LPS), aiming to reveal previously unappreciated biological changes and to investigate how a transcription factor differentiated embryonic chondrocytes 2 (Dec2)‐deficient environment influences the function of autophagy in nflamed human PDLFs.

**Methods:**

A Dec2‐deficient (*Dec2*KO) experimental periodontal inflammation mouse model and treatment with *P. gingivalis* LPS were employed to examine the role of autophagy in PDLFs using hematoxylin and eosin staining and immunohistochemistry in vivo. A Dec2 small interfering RNA (siRNA) was used to modulate autophagy, and the effect of autophagy on the Dec2 pathway was explored using real‐time polymerase chain reaction and western blot analysis in vitro.

**Results:**

LPS‐treated human PDLFs (HPDLFs) induced autophagy, as demonstrated by the enhanced levels of microtubule‐associated protein 1 light chain 3‐II (LC3‐II) and the induction of ATG5, Beclin1, and Dec2. Compared with a scrambled siRNA, a Dec2 siRNA triggered the detrimental influences of LPS and markedly enhanced autophagy expression in inflamed HPDLFs. The expression of phosphorylated ERK was increased and levels of phosphorylated mammalian target of rapamycin (mTOR) were decreased after exposure to LPS in Dec2 siRNA transfected HPDLFs. The *Dec2*KO model exhibited that *P. gingivalis* in Dec2 deficient conditions increases the inflammation of PDLFs by regulating autophagy.

**Conclusions:**

These results demonstrate that a Dec2 deficiency can alleviate LPS‐induced inflammation via the ERK/mTOR signaling pathway by regulating autophagy, conceivably delivering a novel approach for the detection of periodontal treatments.

## Graphical abstract

Dec2 was found in this study to be part of the host defense against microbial challenge in the periodontium, and thus our findings further underline the critical role of autophagy in periodontal diseases.

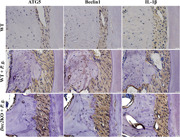

## 1 INTRODUCTION

Autophagy is a molecular process integral to the maintenance of intracellular homeostasis^1^ that is usually triggered under conditions of physiological or pathological stress and acts as an important adaptation for survival. Autophagy can initiate cell protective and survival mechanisms or can cause cell death depending on the severity of stress.^2^ Autophagy can be due to stimuli such as starvation, infection, hypoxia, or an adverse environment, which can further initiate various molecular pathways (mTOR, PI3K‐III/Beclin1, etc.). The role of the autophagy pathway has also been established in the health‐related balance of immunity and inflammation.^3^ Autophagy‐related genes (ATGs) such as Beclin1, microtubule‐associated protein 1 (MAP‐1), and light chain 3 (LC3) play significant roles in these biological processes.^4^ LC3‐II has been shown to influence the autophagy substrate selection and the generation of autophagosomes. Due to this essential role, LC3‐II has been widely used as a marker of autophagosomes.^5^

The tooth is supported by an assembly of tissues called the periodontium, which is composed of the alveolar bone proper, the cementum, and the periodontal ligament (PDL). Among those structures, the PDL is the only nonmineralized connective tissue that lies between the tooth's root (cementum) and the bone.^6^ Periodontal disease is characterized by a chronic inflammation initiated by the colonization of micro‐organisms that results in progressive cellular damage, which manifests clinically as gingival recession, attachment loss, and loss of the tooth/teeth.^7^, ^8^ Bacteria and their products such as lipopolysaccharide (LPS) have been found to promote the secretion of inflammatory cytokines and to reduce cell survival. Those cytokines are known to cause the immune cell‐mediated destruction of collagen fibers and the resorption of alveolar bone.^9^

A role of inflammation promotors in PDL cells has also been implicated in the pathogenesis of periodontitis, which can be influenced by their autophagy.^10^, ^11^ The ubiquitous role of autophagy has also been researched and established in the innate and adaptive immune system along with the pathogenesis of several inflammatory diseases including periodontitis.^12^, ^13^ The increased expression of molecular markers related to ATGs, such as LC3, has been observed in mononuclear cells of peripheral blood from patients with periodontal disease. This further suggests the involvement of autophagy in periodontitis.^11^, ^14^ Clinical studies have shown that the protection of PDL stem cells against apoptosis in an inflammatory microenvironment is related to autophagy.^11^ High levels of autophagy markers have been detected in resorption lacunae of alveolar bones from animal models of periodontitis.^15^ This was also seen to facilitate osteoclastogenesis and osteoclastic bone resorption in RAW264.7 cells.^16^

The role of the transcription factor differentiated embryonic chondrocytes 2 (Dec2) in immune regulation is significant. Th2 cells have been shown to secrete interleukins such as interleukin‐4 (IL‐4), IL‐5, IL‐10, and IL‐13, which have a protective role by inhibiting Th1 cell responses. An immune regulatory ability of Dec2 has also been established based on its preferential expression in Th2 cells, especially in the late phase of the differentiation of helper T cells to Th2 cells. This has also been found to promote the production of Th2‐type cytokines.^17^

Since autophagy mechanisms are seen in most of the cellular stress‐response pathways and since PDL fibroblasts (PDLFs) comprise the majority of cells in PDL connective tissues with a role in maintaining the normal function, protection, and repair,^1^ we speculated that Dec2 may affect the pathogenesis of the inflammation of PDL tissues by altering the normal biological behavior of PDLFs. In this study, we developed a *Dec2*‐deficient mouse strain on a C57BL/6J background and refer to it as *Dec2*KO. We further demonstrate that PDLFs cultured in the presence of LPS show higher levels of autophagy and proinflammatory cytokines than the controls. An experimental mouse periodontitis model further confirmed that a Dec2 deficiency triggers inflammation and autophagy in PDLFs, thus resulting in the destruction of periodontal tissue.

## 2 METHODS

### 2.1 Cell culture

Human PDL fibroblasts (HPDLFs) were obtained from Lonza and were cultured with SingleQuots Supplements (CC‐4181, Insulin, hFGF‐β, GA‐1000, and fetal bovine serum) at 37°C. HPDLFs were subcultured when they became 70%–80% confluent and cells at passages 8–9 were used for these experiments. The cells were incubated in the presence or absence of 1 µg/ml *Porphyromonas gingivalis* LPS (LPS‐PG Ultrapure; InvivoGen) to induce an inflammatory response for 24 h.

### 2.2 Small interfering RNA (siRNA) transfection

A scrambled siRNA (negative control) or a Dec2 siRNA were transfected into 1.0 × 10^5^ HPDLFs using RNAiMAX (Thermo Fisher Scientific). The HPDLFs were harvested 48 h after transfection and were analyzed by real‐time polymerase chain reaction (RT‐PCR) and western blot analysis.

### 2.3 RT‐PCR

Total RNAs of HPDLFs were extracted using a miRNeasy Mini Kit (Qiagen). One microgram of each RNA was transcribed to complementary DNA and TaqMan probes (IL‐1β, Dec2, ATG5, Beclin1, LC3‐I, LC3‐II, and ACTB; Thermo Fisher Scientific) were used.

### 2.4 Western blot

Twenty micrograms of each protein extract were electrophoresed per lane on 10% or 15% gels (Wako) and were then transferred to polyvinylidene difluoride membranes. The membranes were blocked with 5% skim milk or 5% bovine serum albumin for 1 h and were then incubated with antibodies to Dec2 (Hiroshima University), IL‐1β, p‐ERK1/2, ERK1/2, p‐mTOR, mTOR, p70s6K, p‐4EBP1, glyceraldehyde 3‐phosphate dehydrogenase (Cell Signaling Technology), and ATG5, Beclin1, LC3‐I/II, p62 (Abcam) overnight at 4°C. The bound antibodies were anticipated using an enhanced chemiluminescence system (GE Healthcare) and ImageJ software was used to measure band intensities.

### 2.5 Animals

*Dec2*KO mice were generated like *Dec1*KO mice as described previously.^18^ The entire coding region was replaced with a Neo cassette. The resulting chimeric mice were backcrossed to a C57BL/6J (wild‐type [WT]) background for three generations. The breeding and animal care were carried out by trained professionals at Oriental Yeast Co., according to the standard protocols until arrival at our animal facility. WT (n = 12) and *Dec2* knockout (*Dec2*KO, n = 12) male mice aged 12 weeks old were maintained in a pathogen‐free environment and were used as a periodontitis model as previously described.^18^ The mice were killed after 30 days and 4% paraformaldehyde (Wako) was used to fix the jaws for immunohistochemistry. A mixture of midazolam at a dose of 4 mg/kg, butorphanol tartrate at a dose of 5 mg/kg (0.1 ml/10 g body weight), and medetomidine hydrochloride at a dose of 0.3 mg/kg was used to sacrifice the mice by cervical dislocation under deep anesthesia. All the animal experiments were conducted under the approval of the Animal Ethics Committee of Kanagawa Dental University and appliance with guidelines.

### 2.6 Immunohistochemistry

Antigen retrieval and peroxidase blocking of specimens were performed according to standard protocols, followed by incubation with antibodies to ATG5, Beclin1, HIF‐1α (Abcam), and 8‐OHdG (JaICA) overnight at 4°C. The specimens were subjected to incubation with secondary antibodies (Nichirei Bioscience) for 30 min followed by the 3,3’‐diaminobenzidine staining.

### 2.7 Statistical analysis

Independent two‐tailed Student's *t* test or analysis of variance were used for the analysis of results via SPSS 16.0. A *p* < .05 is considered significant.

## 3 RESULTS

### 3.1 Dec2 is involved in LPS‐induced autophagy

**Figure 1:**
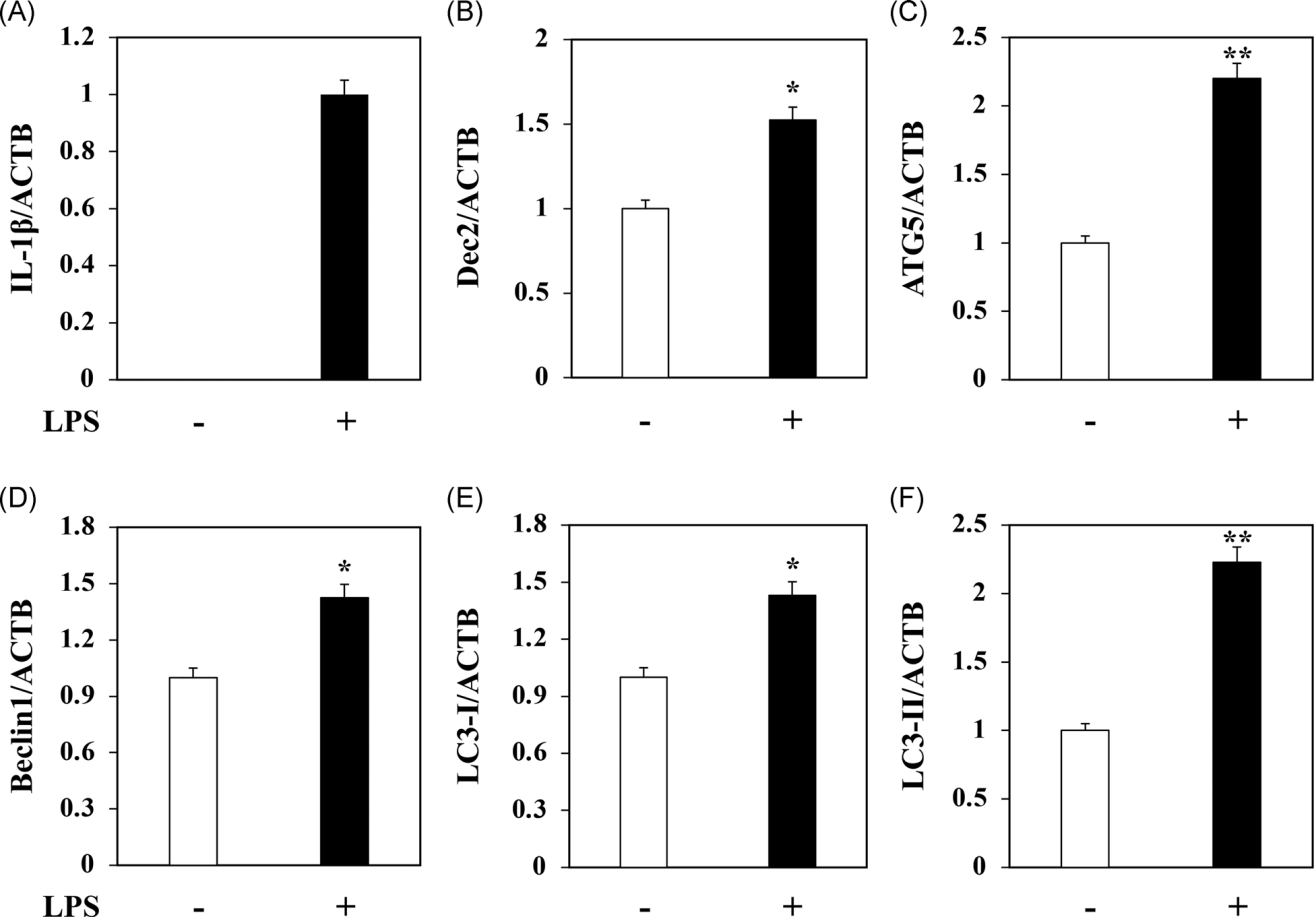
Dec2 is involved in LPS‐induced autophagy. RT‐PCR analysis showing that: (A) IL‐1β is induced by treatment with 1 µg/ml *Porphyromonas gingivalis* LPS. (B) The high expression level of IL‐1β is consistent with the elevated expression level of Dec2. (C–F) The autophagy markers, ATG5, Beclin1, LC3‐I, and LC3‐II, were upregulated by treatment with 1 µg/ml *P. gingivalis* LPS. *p < .05, **p < .01 are represented means ± *SD*. Three independent experiments are performed for all the results. Dec2, differentiated embryonic chondrocytes 2; IL, interleukin; LC3, light chain 3; LPS, lipopolysaccharide; RT‐PCR, real‐time polymerase chain reaction; *SD*, standard deviation

To understand the interaction between autophagy and the transcription factor Dec2, we treated HPDLFs with 1 µg/ml *P. gingivalis* LPS for 24 h. RT‐PCR analysis showed that LPS dramatically induced the expression of IL‐1β (Figure 1A). Interestingly, the increased expression of IL‐1β was accompanied by the elevated expression level of Dec2 (Figure 1B, *p* < .05). The autophagy markers, ATG5 (Figure 1C, *p* < .01), Beclin1 (Figure 1D, *p* < .05), LC3‐I (Figure 1E, *p* < .05), and LC3‐II (Figure 1F, *p* < .01) were also significantly upregulated by treatment with 1 µg/ml *P. gingivalis* LPS.

### 3.2 A Dec2 deficiency facilitates inflammation by upregulating autophagy

**Figure 2:**
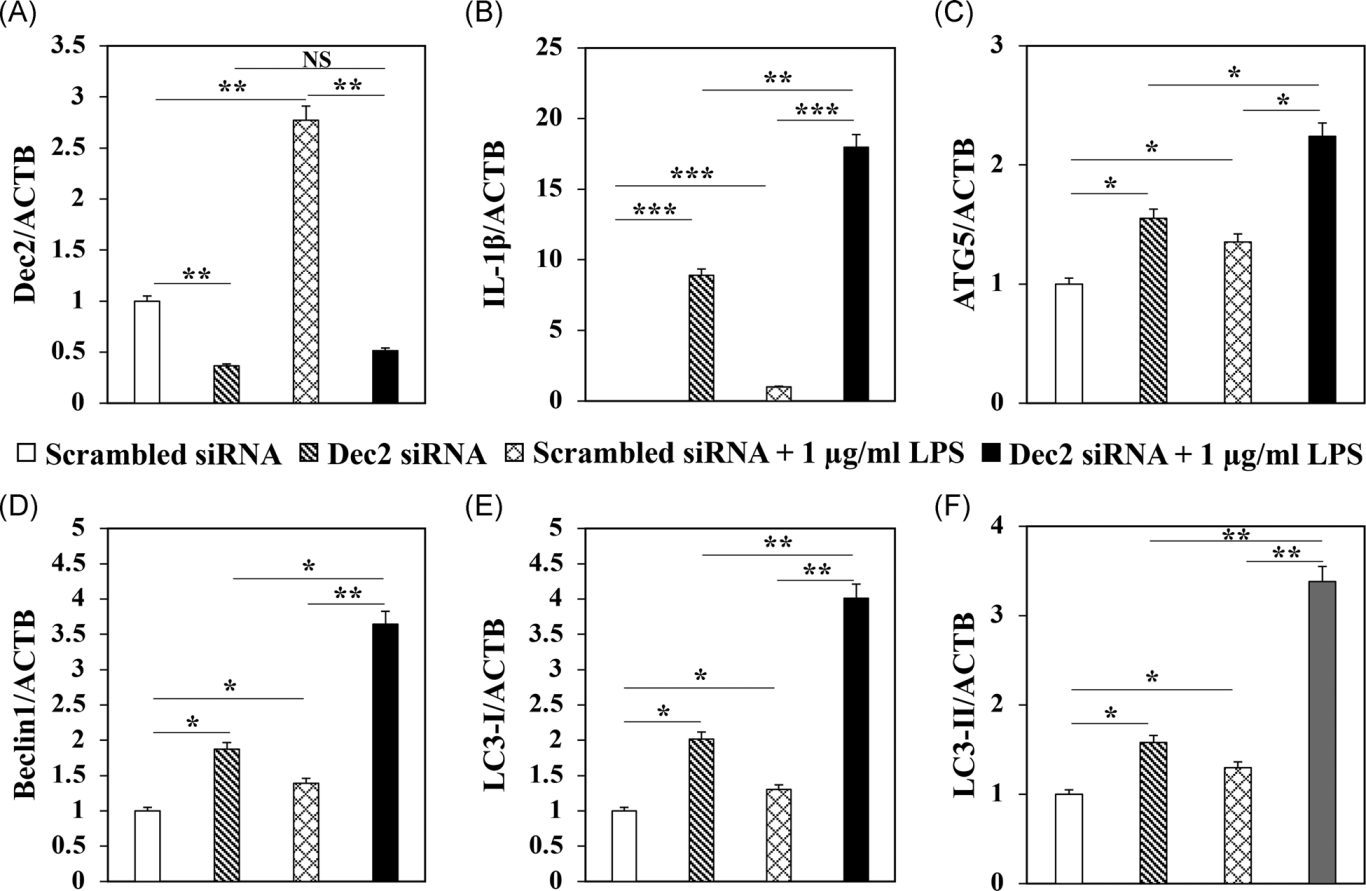
A Dec2 deficiency facilitates inflammation by upregulating autophagy. RT‐PCR analysis showing that: (A) Dec2 was suppressed after transfection with a Dec2 siRNA. (B) The Dec2 deficiency‐induced IL‐1β, moreover, treatment with 1 µg/ml *Porphyromonas gingivalis* LPS facilitated inflammatory activity. (C–F) The expression levels of ATG5, Beclin1, LC3‐I, and LC3‐II were also increased with the Dec2 deficiency, and treatment with LPS magnified the impact of Dec2 knockdown. *p < .05, **p < .01, ***p < .001 are represented means ± *SD*. Three independent experiments are performed for all the results. Dec2, differentiated embryonic chondrocytes 2; IL, interleukin; LC3, light chain 3; LPS, lipopolysaccharide; RT‐PCR, real‐time polymerase chain reaction; siRNA, small interfering RNA; *SD*, standard deviation

To verify the regulatory effect of Dec2 on autophagy‐mediated inflammation, HPDLFs were transfected with a Dec2 siRNA. RT‐PCR analysis showed that levels of Dec2 were significantly suppressed after transfection with the Dec2 siRNA (Figure 2A, *p* < .01). These results revealed that the Dec2 deficiency dramatically induced the expression of IL‐1β, and moreover, treatment with 1 µg/ml *P. gingivalis* LPS facilitated the inflammatory activity (Figure 2B, **p < .01, ***p < .001). The expression levels of ATG5 (Figure 2C, *p* < .05), Beclin1 (Figure 2D, *p < .05, **p < .01), LC3‐I (Figure 2E, *p < .05, **p < .01), and LC3‐II (Figure 2F, *p < .05, **p < .01) were also significantly increased by the Dec2 deficiency, and treatment with LPS magnified the impact of the Dec2 knockdown.

### 3.3 A Dec2 deficiency activated autophagy‐mediated inflammation via the ERK/mTOR pathway

**Figure 3:**
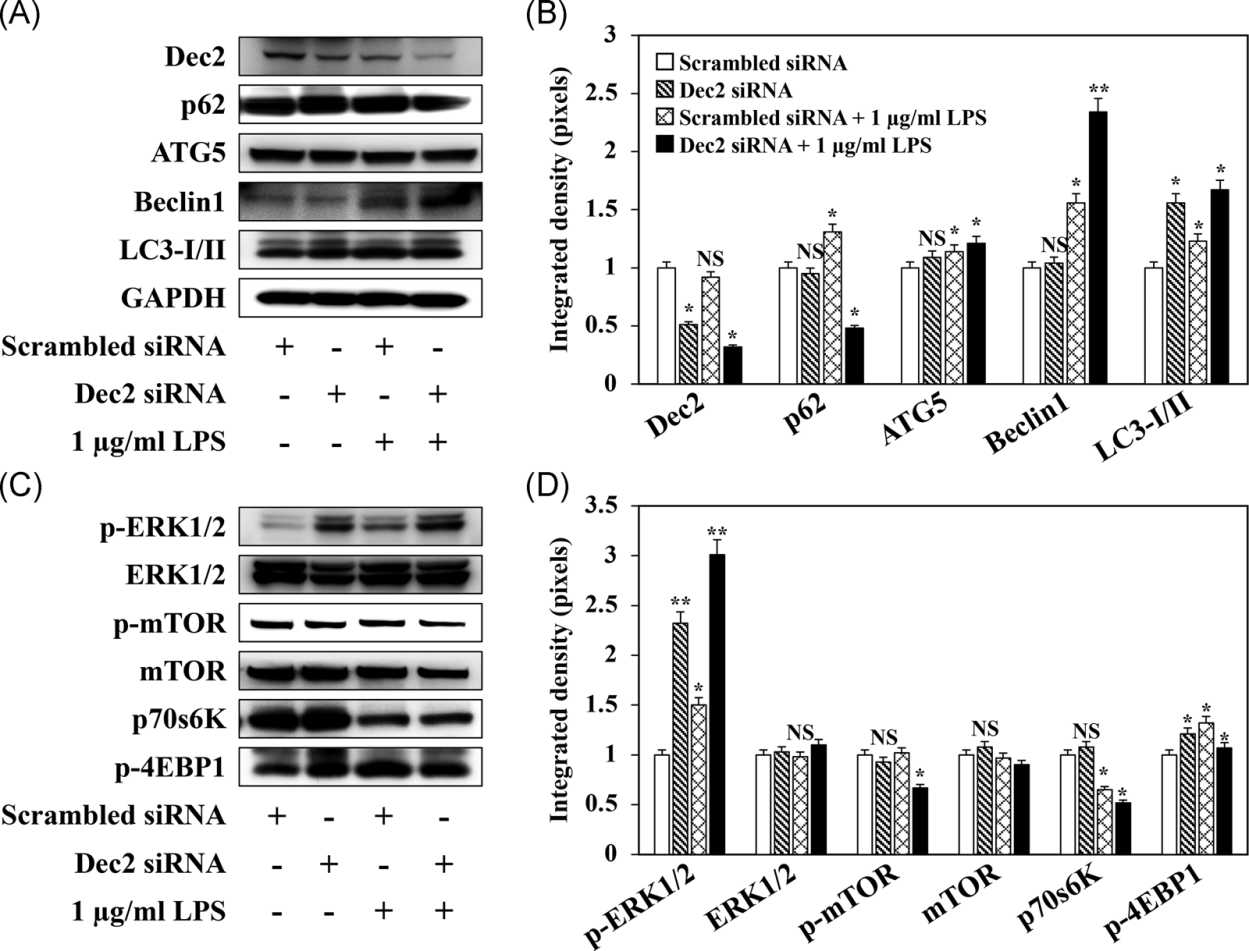
A Dec2 deficiency activates autophagy‐mediated inflammation via the ERK/mTOR pathway. (A,B) Western blot confirming that the Dec2 deficiency significantly inhibited p62 expression, induced the expression of autophagy markers, and treatment with 1 µg/ml *Porphyromonas gingivalis* LPS amplified this effect. (C,D) Western blot showing that the lack of Dec2 enhanced the phosphorylation of ERK1/2. Phosphorylated ERK1/2 suppressed mTOR phosphorylation and the expression of p70s60K and p‐4EBP1. *p < .05, **p < .01, ***p < .001 are represented means ± *SD*. Three independent experiments are performed for all the results. Dec2, differentiated embryonic chondrocytes 2; GAPDH, glyceraldehyde 3‐phosphate dehydrogenase; LC3, light chain 3; LPS, lipopolysaccharide; siRNA, small interfering RNA; *SD*, standard deviation

Consistent with the results above, western blots confirmed that the Dec2 deficiency (Figure 3A,B, *p* < .05) significantly inhibited the expression of p62 (Figure 3A,B, *p* < .05), and induced expression of the autophagy markers, ATG5 (Figure 3A,B, *p* < .05), Beclin1 (Figure 3A,B, *p < .05, **p < .01), and LC3‐I/II (Figure 3A,B, *p* < .05). Treatment with *P. gingivalis* LPS amplified that effect. Furthermore, the lack of Dec2 enhanced the phosphorylation of ERK1/2 (Figure 3C,D, *p < .05, **p < .01). The phosphorylated ERK1/2 suppressed the phosphorylation of mTOR (Figure 3C,D, *p* < .05), which inhibited the mTOR pathway by downregulating p70s60K (Figure 3C,D, *p* < .05) and p‐4EBP1 (Figure 3C,D, *p* < .05). The inactivated mTOR led to an induction of autophagy with the Dec2 deficiency and treatment with *P. gingivalis* LPS.

### 3.4 Dec2 suppresses autophagy in periodontitis

**Figure 4:**
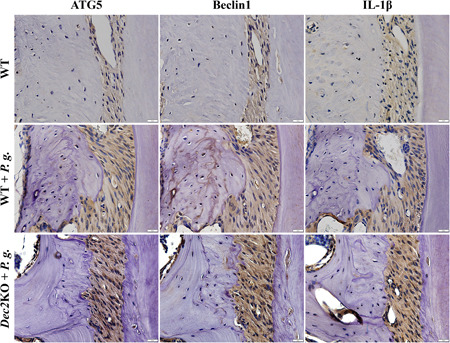
Dec2 suppresses autophagy in periodontitis. The expression of ATG5 and Beclin1 were highly upregulated in the PDL of *Dec2*KO mice compared with WT mice after treatment with *Porphyromonas gingivalis*. The induced autophagy led to an overexpression of IL‐1β in *Dec2*KO mice compared with WT mice. Original magnification: ×60, scale bars = 20 μm. All results are representative of at least three independent experiments. Dec2, differentiated embryonic chondrocytes 2; IL, interleukin; PDL, periodontal ligament; *SD*, standard deviation; WT, wild‐type

To confirm the effect of Dec2 on autophagy during inflammation, we used two periodontitis models. The expression of ATG5 and Beclin1 were highly upregulated in *Dec2*KO mice compared with WT mice after treatment with *P. gingivalis* (Figure 4). The induced autophagy led to an overexpression of IL‐1β in *Dec2*KO mice compared with WT mice. The expression level of the hypoxia marker HIF‐1α and the oxidative stress marker 8‐OHdG were both increased with the Dec2 deficiency during periodontitis (Figure S1).

## 4 DISCUSSION

The present study determined the functions and mechanisms of Dec2 on the behavior of PDLFs using in vitro experiments. The results demonstrate that transfection with a Dec2 siRNA significantly increased the autophagy of HPDLFs induced by *P. gingivalis* LPS. Further, Dec2 siRNA knockdown stimulated the LPS‐induced production of IL‐1β and treatment with *P. gingivalis* LPS combined with a Dec2 deficiency activated cell autophagy and the autophagy‐related marker mTOR‐4EBP1, which are hallmarks of the cellular autophagy signaling pathway. Finally, we demonstrated that Dec2 plays an essential role in PDLF inflammation that depends on cell autophagy. Thus, these findings implicate a Dec2 deficiency in cell homeostasis through modulation of the autophagy pathways and the production of proinflammatory cytokines. Additionally, these results may suggest new approaches for the management of the periodontal disease.

Autophagy has been shown to be a protective mechanism in eukaryotic cells and to be closely related to the fate of cells in periodontitis^19^ and several other inflammatory diseases. Autophagy has beneficial effects on PDLFs by protecting them from inflammation‐induced apoptosis, which is causally related to the presence of excessive environmental stresses.^11^, ^20^ Disturbances in this balance have been found to be associated with infective stimuli, neuromuscular disorders, hepatic diseases, and neoplasia.^21^ The survival of cells is adversely affected by an excess of autophagic stimuli as well. The present study was aimed to explore and identify potential molecular targets of autophagy in PDLFs so that future research can be directed towards developing a better understanding of its effects on periodontal health. Similarly, there has been little or no work relating autophagy and Dec2 with the etiopathogenesis of periodontal diseases. We observed that the autophagy‐related proteins LC3‐II/I, ATG5, and Beclin1 had a significantly increased expression after the PDLFs were subjected to challenge with *P. gingivalis* LPS. We used 1 μg/ml *P. gingivalis* LPS to stimulate HPDLF inflammation in this study since CellTiter 96 AQueous assay showed no cell toxicity present in LPS‐treated cells (data not shown). Dec2 expression increased significantly in response to *P. gingivalis* LPS over a period of 24 h. Inhibiting Dec2 worsened the autophagy as reflected by an unusual increase in the Beclin1 expression and the ratio of LC3‐II/LC3‐I in the LPS‐treated group compared with the controls. However, a decreased level of p62 was observed in these cells. These findings prove that a Dec2 deficiency promotes autophagy in *P. gingivalis* LPS‐treated HPDLFs. The Dec2 deficiency‐induced autophagy and signaling pathways also exhibited an increased expression in the LPS‐treated group. Hence, a regulatory role for Dec2 in the improvement of inflammation‐induced PDLF autophagy, as seen in periodontitis, has been strongly established. However, there was no significant difference between control and *P. gingivalis* LPS‐treated group in human gingival fibroblasts (HGFs) (data not shown).

The pivotal contributions of autophagy in immunity are related to the autonomous control of cell viability, tissue/organ damage, and the increased production of proinflammatory mediators.^22^, ^23^ Changes in the expression of factors associated with autophagy due to inflammatory and microbial insults have also been established. Similarly, IL‐1β stimulated the engagement of inflammatory cells, and the production of enzymes has been implicated in the pathogenesis of periodontal diseases.^24^, ^25^ A Dec2 deficiency was also found to increase the *P. gingivalis* LPS‐induced upregulation of the inflammatory cytokine IL‐1β. In the *Dec2*KO mouse model, an increased accumulation of dental plaque due to *P. gingivalis* caused substantial periodontal inflammation and bone loss. Interestingly, our results highlight that the initiation of periodontitis resulted in the increased expression level of IL‐1β protein in the 30 days study period. The elevated production of tumor necrosis factor‐α, IL‐1β, and IL‐6 have also been observed in patients with periodontal disease, which further leads to PDL destruction and attachment loss.^26^, ^27^ We failed to detect the functional binding sites between Dec2 and IL‐1β, suggesting an indirect response (data not shown). In the future, this experimental periodontitis model can be used to evaluate the regulation of Dec2 with other cytokines.

The MEK/ERK signaling pathway has been made a significant contribution to cellular autophagy via the modulation of LC3 expression.^28^ We observed that LC3‐II accumulation and p62 reduction can be related to the activity of this signaling pathway. Treatment with *P. gingivalis* LPS significantly increased the expression of p‐ERK1/2, the conversion of LC3 and the decrease in p62 expression in HPDLFs. Moreover, the Dec2 siRNA knockdown caused a substantial increase of LC3‐II and the decreased p62 expression in *P. gingivalis* LPS‐treated HPDLFs. Our findings indicate that the autophagy in *P. gingivalis* LPS‐treated HPDLFs is related to activation of the ERK signaling pathway.

mTOR is another crucial target in the induction of autophagy. Inflammation enhanced autophagy levels of HPDLFs in periodontitis, which accordingly regulated mTOR and ERK, the two important signaling pathways. A heart failure model showed explicitly a balance in the mechanism of apoptosis/autophagy via mTOR.^29^ In this study, Dec2 inhibited autophagy, first by phosphorylating mTOR and then by activating 4EBP1. Therefore, the downregulation of mTOR by Dec2 siRNA and treatment with *P. gingivalis* LPS stimulation demonstrates a transcriptional regulation of autophagy. Therefore, these data reveal that Dec2 regulates cell inflammation mediating the autophagy pathway, possibly providing a new perspective for the periodontal treatment.

LC3 plays a pivotal role in the fusion step of autophagy. The autophagy proteins LC3I/II and Beclin1 are imperative in the formation of autophagosome. An experimental periodontitis mouse model exhibited increased LC3‐II and Beclin1 expression.^15^ Furthermore, periodontitis patients also showed a similar trend in inflamed PDL.^11^ The ratio of LC3‐II/LC3‐I and the number of autophagic HGFs were increased after treatment with LPS.^30^ Our study also showed a notable increase in ATG5, Beclin1, and LC3‐II/LC3‐I expression in inflammatory HPDLFs compared with their minimal expression in control HPDLFs. After 24 h of LPS stimulation in Dec2 deficient cells, the changes in gene expression are mainly in support of autophagy upregulation in HPDLFs, except for the downregulation of p62. These data also indicate that PDLFs responding to LPS stimulation were able to significantly increase autophagy and a Dec2 deficiency leads to substantial changes, showing a tendency towards the robust induction of autophagy.

PDLFs select and attract inflammatory cells, causing them to migrate to the bone surface in the pathological process of periodontitis.^7^ Periodontal patients revealed the increased expression of autophagy markers in the peripheral blood and also the increased autophagosome production in PDL.^11^, ^14^ Our results are in line with these studies suggesting the involvement of autophagy in periodontal inflammation.

## 5 CONCLUSION

Activation of transcription factors and thereby the changes in gene expression is necessary to maintain or restore homeostasis to neutralize uncertain modifications in the environment. Thus, an established in vivo animal model is imperative to acclaim in vitro cell culture experiments to improve the perception of cellular and molecular regulation of autophagy. Dec2 might relate to host immune defense response against microbial pathogens in the periodontium, and thus our results also emphasize the importance of autophagy in periodontal inflammation. A limitation of this study is that we studied only the phenomena of *P. gingivalis*, autophagy and Dec2 in PDLFs and thus the molecular function involved and whether Dec2 is also expressed by other periodontal tissue‐resident cells should be explored in future studies. Our approach exemplifies a unique target for Dec2 regulation in periodontitis. Suppressing autophagy by addressing Dec2 might debilitate the aberrant inflammation that occurs in PDLFs.

## CONFLICT OF INTERESTS

The authors declare that there are no conflict of interests.

## AUTHOR CONTRIBUTIONS

*Contributed to the study design and conceive*: Shunichi Oka. *Contributed to the experiment performance and manuscript writing*: Xiaoyan Li. *Contributed to the animal experiments and data analyze*: Fengzhu Zhang and Chongchong Chen. *Contributed to the manuscript and data critical review*: FuyukiSato, Nitesh Tewari, Makoto Makishima, Liangjun Zhong, and Yi Liu. *Contributed to the study conceive, animal experiments, data analyze, and manuscript writing*: Ujjal K. Bhawal. The final manuscript was approved by all the authors.

## Supporting information

Supporting information.

Supporting information.

## Data Availability

The data supporting the findings of this study are available at https://doi.org/10.6084/m9.figshare.13109969.v1.
