# Supplementary figures and images for: Dec2 attenuates autophagy in inflamed periodontal tissues
Source: Immun Inflamm Dis. 2020 Dec 3;9(1):265–73. doi: 10.1002/iid3.389 (PMC7860609; doi:10.1002/iid3.389)

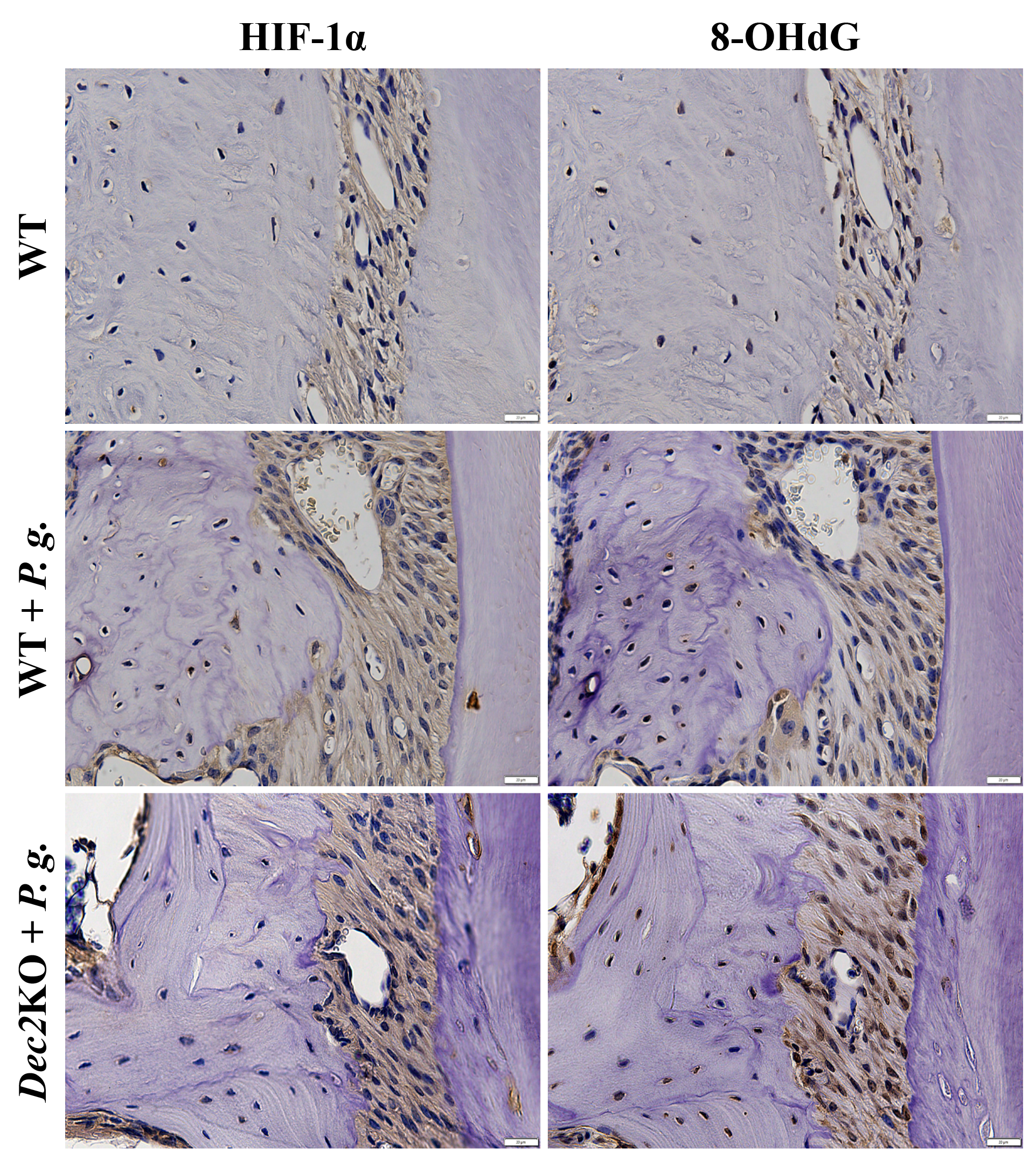

Supplement: Supplementary file 1 — Supporting information. [file IID3-9-265-s001.tif]
