# Supplementary material for: Dec2 attenuates autophagy in inflamed periodontal tissues
Source: Immun Inflamm Dis. 2020 Dec 3;9(1):265–73. doi: 10.1002/iid3.389 (PMC7860609; doi:10.1002/iid3.389)
Supplement: Supplementary file 2 — Supporting information. [file IID3-9-265-s002.docx]

**FIGURE S1** Dec2 deficiency induced hypoxia and ROS in periodontal tissues. The expression level of the hypoxia marker HIF-1α and the oxidative stress marker 8-OHdG were both increased with the Dec2 deficiency during periodontitis. Original magnification: 60x, scale bars▒=▒20 μm. All results are representative of at least three independent experiments.
